# Use of synthetic population to assess alcohol abuse in small areas

**DOI:** 10.1590/1980-549720260013

**Published:** 2026-04-10

**Authors:** Crizian Saar Gomes, Regina Tomie Ivata Bernal, Larissa Fortunato Araújo, Celso Renato França, Samuel Norberto Alves, Juliana Bottoni de Souza, Flora Vitória Serena Oliveira Baldi, Marcos André Gonçalves, Jussara Marques Almeida, Deborah Carvalho Malta

**Affiliations:** I Universidade Federal de Minas Gerais, Post-Graduation Program in Public Health, School of Medicine - Belo Horizonte (MG), Brazil.; II Universidade Federal de Minas Gerais, Post-Graduation Program in Nursing, School of Nursing - Belo Horizonte (MG), Brazil.; III Universidade Federal do Ceará, Health Sciences Center, Department of Community Health - Fortaleza (CE), Brazil.; IV Universidade Federal de Minas Gerais, Post-Graduation Program in Computer Science, Institute of Exact Sciences - Belo Horizonte (MG), Brazil.; V Universidade Federal de Minas Gerais, School of Medicine - Belo Horizonte (MG), Brazil.; VI Universidade Federal de Minas Gerais, School of Nursing - Belo Horizonte (MG), Brazil.; VII Universidade Federal de Minas Gerais, Institute Exact Sciences, Department of Computer Science - Belo Horizonte (MG), Brazil.; VIII Universidade Federal de Minas Gerais, School of Nursing, Department of Maternal-Child Nursing and Public Health - Belo Horizonte (MG), Brazil.

**Keywords:** Abusive alcohol consumption, Generative adversarial networks, Synthetic population, Population surveys

## Abstract

**Objective:**

This study evaluated the use of synthetic populations, generated by conditional generative adversarial networks (cGAN), to estimate abusive alcohol consumption in small areas of Belo Horizonte (MG).

**Methods:**

Data from the Vigitel survey from 2006 to 2018 were used. Sociodemographic and lifestyle variables were considered, and the synthetic records were assigned to nine vulnerability clusters.

**Results:**

The inclusion of synthetic data reduced standard errors and narrowed confidence intervals, especially in more vulnerable areas. Higher prevalence was observed in less vulnerable areas.

**Conclusions:**

The approach proved promising for overcoming representativeness limitations and enhancing risk factor monitoring, but it still depends on the quality of the original data, may introduce biases, and requires external validation and cautious use.

## INTRODUCTION

The analysis of risk factors in small areas is essential for understanding social inequalities in health and for supporting the planning of local public policies. Brazil, despite advances in surveillance, still faces difficulties in producing valid estimates in small areas because of the high costs and challenges in data collection, which make the implementation of representative surveys unfeasible. The Surveillance System of Risk and Protective Factors for Chronic Diseases by Telephone Survey (Vigitel), an annual telephone survey covering the state capitals, is one of the main sources of monitoring, but its dependence on landlines limits representativeness in more vulnerable groups, such as low-income populations and residents of peripheral areas^1^.

These limitations have stimulated the development of alternative methodologies to increase accuracy in small areas. The international literature suggests the use of approaches such as Bayesian models, multilevel models, and small area estimation techniques^2^.

More recently, innovations in artificial intelligence, such as conditional generative adversarial networks (cGANs), have been applied to generate realistic synthetic populations, enabling simulations that expand the analytical capacity in contexts where real data are scarce, confidential, or difficult to obtain^3^. This study aimed to evaluate the use of synthetic populations, generated by cGANs, to assess alcohol abuse in small areas of Belo Horizonte (MG), exploring its methodological potential and discussing its implications.

## METHODS

This was an ecological study conducted with Vigitel data for Belo Horizonte (MG), between 2006 and 2018. The addresses of the interviewees, provided by the operators, were georeferenced and linked to the National Register of Addresses for Statistical Purposes, allowing association with census tracts^2^.

Abusive alcohol consumption was defined as consuming four or more drinks on one occasion for women and five or more for men^4^. Nine vulnerability clusters (LO-0, LO-1, MD-0, MD-1, HI-0, HI-1, HI-2, VH-0, VH-1) were considered “small areas,” derived from the four categories of the Health Vulnerability Index (HVI) (Low, Medium, High, and Very High). This regrouping strategy was proposed in a previous study^5^ to reduce the variability of HVI. Synthetic populations were generated by cGAN networks implemented in PyTorch, following the classic GAN architecture, where the generator and discriminator are jointly optimized in an adversarial scenario. Training occurred over 1,024 epochs, with a batch size of 500, a learning rate of 2×10⁻⁴, and the Adam optimizer (default parameters). The stopping criterion was based solely on the number of epochs, without early stopping, and the adopted loss function was the canonical adversarial loss (minimax log loss). The variables used were sociodemographic and lifestyle variables (e.g., sex, age, race/skin color, education level, and daily fruit consumption), selected for their epidemiological relevance and availability in the database. Between 5,000 and 100,000 synthetic records were generated per cluster, from which subsamples of 700 individuals were selected (defined on the basis of the stabilization of the standard error at ~1%) and incorporated, using supervised models such as XGBoost, into the Vigitel database in the high and very high vulnerability clusters, totaling 3,500 interviews per period (Table 1). The similarity between real and synthetic distributions was determined using the KSComplement index. The prevalence and 95% confidence interval (95%CI) were estimated by cluster and period (2006-2009; 2010-2013; 2014-2018), considering post-stratification weights. Differences between clusters within the same period were assessed by the non-overlapping of the 95%CIs.

The study was approved by the Research Ethics Committee of the Federal University of Minas Gerais (Opinion No. 6.538.883).

**Table 1.** Sample size of the Surveillance System for Risk and Protective Factors for Chronic Diseases by Telephone Survey (Vigitel) and the synthetic population by cluster and by period.

Cluster	2006 to 2009			2010 to 2013			2014 to 2018		
	Real population	Synthetic population	Total	Real population	Synthetic population	Total	Real population	Synthetic population	Total
1-LO-0	1,723	-	1,723	1,453	-	1,453	1,570	-	1,570
2-LO-1	1,228	-	1,228	1,112	-	1,112	946	-	946
3-MD-0	1,477	-	1,477	1,297	-	1,297	1,485	-	1,485
4-MD-1	1,547	-	1,547	1,628	-	1,628	1,721	-	1,721
5-HI-0	324	700	1,024	356	700	1,056	376	700	1,076
6-HI-1	130	700	830	158	700	858	197	700	897
7-HI-2	601	700	1,301	683	700	1,383	763	700	1,463
8-VH-0	93	700	793	104	700	804	107	700	807
9-VH-1	199	700	899	242	700	942	244	700	944
Total	7,322	3,500	10,822	7,033	3,500	10,533	7,409	3,500	10,909

LO: low; MD: medium; HI: high; VH: very high;

## Data Availability Statement:

Data are available upon request from the corresponding author.

## RESULTS

The prevalence of alcohol abuse was consistently higher in the less vulnerable clusters across all periods analyzed, both in the real data (T1 28.2%; 95%CI 25.1-31.3; T2 26.2%; 95%CI 23.0-29.4; T3 27.0%; 95%CI 22.9-31.0) and in the synthetic data (T1 27.4%; 95%CI 24.7-30.0; T2 27.7%; 95%CI 24.7-30.7; T3 26.5%; 95%CI 22.6-30.5) (Table 2).

**Table 2.** Prevalence of abusive alcohol consumption in the nine vulnerability clusters for the periods 2006 to 2009 (T1), 2010 to 2013 (T2) and 2014 to 2018 (T3).

	Real population						Real+synthetic population					
	T1 % (95%CI)	SE	T2 % (95%CI)	SE	T3 % (95%CI)	SE	T1 % (95%CI)	SE	T2 % (95%CI)	SE	T3 % (95%CI)	SE
LO-0	23.5 (21.0-25.9)	1.2	23.9 (21.2-26.7)	1.4	24.1 (21.2-26.9)	1.5	23.3 (21.12-25.4)	1.1	24.1 (21.51-26.6)	1.3	24.0 (21.3-26.7)	1.4
LO-1	28.2 (25.1-31.3)	1.6	26.2 (23.0-29.4)	1.6	27.0 (22.9-31.0)	2.1	27.4 (24.7-30.0)	1.4	27.7 (24.7-30.7)	1.5	26.5 (22.6-30.5)	2.0
MD-0	20.3 (17.7-22.9)	1.3	20.7 (18.0-23.4)	1.4	21.7 (18.9-24.5)	1.4	19.5 (17.3-21.6)	1.1	21.8 (19.3-24.3)	1.3	21.7 (19.1-24.3)	1.3
MD-1	20.5 (18.0-22.9)	1.2	18.9 (16.7-21.2)	1.1	20.9 (18.3-23.4)	1.3	20.1 (17.9-22.3)	1.1	19.1 (17.0-21.1)	1.1	20.6 (18.1-23.0)	1.2
HI-0	11.2 (7.4-14.9)	1.9	18.4 (13.5-23.3)	2.5	18.3 (13.1-23.5)	2.6	12.0 (9.8-14.2)	1.1	17.5 (14.8-20.2)	1.4	18.9 (15.9-21.8)	1.5
HI-1	17.2 (9.3-25.1)	4.0	19.6 (11.8-27.5)	4.0	21.6 (14.4-28.9)	3.7	17.8 (14.9-20.6)	1.5	19.1 (15.9-22.3)	1.6	21.3 (18.0-24.6)	1.7
HI-2	17.0 (13.3-20.7)	1.9	15 (11.6-18.4)	1.7	15.2 (11.8-18.5)	1.7	17.6 (15.3-19.9)	1.2	14.6 (12.4-16.8)	1.1	15.2 (12.9-17.5)	1.2
VH-0	15.9 (7.3-24.4)	4.3	20.3 (10.3-30.3)	5.1	9.5 (3.2-15.7)	3.2	15.3(12.4-18.2)	1.5	20.6 (17.3-23.9)	1.7	9.2 (6.7-11.7)	1.3
VH-1	14.5 (8.8-20.2)	2.9	14.0 (9.2-18.8)	2.4	15.2 (9.3-21.1)	3.0	14.3 (11.7-16.9)	1.3	14.9 (12.1-17.7)	1.4	15.2 (12.3-18.0)	1.5

SE: standard error; LO: low; MD: medium; HI: high; VH: very high; 95%CI: 95% confidence interval; KSComplement: 0.96.

The inclusion of synthetic populations in the database reduced standard errors and narrowed confidence intervals, especially in the most vulnerable areas, where the original samples were smaller. For example, in cluster VH-0 and period T1, the 95%CI ranged 7.3-24.4% (SE=4.3) with real data and 12.4-18.2% (SE=1.5) with synthetic data (Table 2). The KSComplement index of 0.96 confirmed high similarity between the observed and synthetic distributions.

## DISCUSSION

The findings reinforce that the use of synthetic populations generated by cGAN can mitigate limitations of population-based surveys by producing more accurate estimates in small areas. The higher consumption of alcoholic beverages in less vulnerable areas is consistent with the national and international literature^4^ ^,^ ^6^ and can be explained by the fact that, in higher-income contexts, there is not only greater financial availability for the purchase of alcoholic beverages, but also a greater supply of commercial establishments and social spaces that encourage drinking. In addition, cultural and behavioral aspects, such as the valuing of social practices linked to alcohol consumption, contribute to reinforcing this pattern. On the other hand, more vulnerable areas may show a lower reported prevalence, but are more exposed to harms resulting from alcohol because of the lower availability of health services and social support.

The use of synthetic data contributed to greater stability of the estimates, reducing variability between periods and proving to be a promising approach to mitigate the scarcity of collected data and reduce distribution and representation biases in small geographical areas.

Despite the methodological advances, the technique should be considered exploratory. Its application depends on the quality of the real data, may introduce biases related to modeling, and requires external validation in different contexts^7^. Ethical issues emerge from the use of synthetic data, including the need for transparency and safeguards against inappropriate uses. When compared to traditional small-area approaches, such as Bayesian and multilevel models, cGANs show competitive and complementary performance^2^ ^,^ ^7^. However, its adoption requires additional studies that evaluate sensitivity, generalization, and replication in other populations.

Thus, it is concluded that synthetic populations represent an innovative resource for health surveillance, but their use must be accompanied by critical assessments, continuous validation, and ethical reflection. This innovation can support the formulation of more precise and equitable public policies, provided it is used responsibly.
