# Supplementary material for: Use of synthetic population to assess alcohol abuse in small areas
Source: Rev Bras Epidemiol. 2026 Apr 10;29:e260013. doi: 10.1590/1980-549720260013 (PMC13078829; doi:10.1590/1980-549720260013)
Supplement: Translation

# Translation: Uso de população sintética para estimativas de consumo abusivo de bebidas alcoólicas em pequenas áreas

**DOI:** 10.1590/1980-549720260013.2

**Authors:** Crizian Saar Gomes, Regina Tomie Ivata Bernal, Larissa Fortunato Araújo, Celso Renato França, Samuel Norberto Alves, Juliana Bottoni de Souza, Flora Vitória Serena Oliveira Baldi, Marcos André Gonçalves, Jussara Marques Almeida, Deborah Carvalho Malta

**Affiliations:** I Universidade Federal de Minas Gerais, Programa de Pós-Graduação em Saúde Pública, Faculdade de Medicina - Belo Horizonte (MG), Brasil.; II Universidade Federal de Minas Gerais, Programa de Pós-Graduação em Enfermagem, Escola de Enfermagem - Belo Horizonte (MG), Brasil.; III Universidade Federal do Ceará, Centro de Ciências da Saúde, Departamento de Saúde Comunitária - Fortaleza (CE), Brasil.; IV Universidade Federal de Minas Gerais, Programa de Pós-Graduação em Ciências da Computação, Instituto de Ciências Exatas - Belo Horizonte (MG), Brasil.; V Universidade Federal de Minas Gerais, Faculdade de Medicina - Belo Horizonte (MG), Brasil.; VI Universidade Federal de Minas Gerais, Escola de Enfermagem - Belo Horizonte (MG), Brasil.; VII Universidade Federal de Minas Gerais, Instituto de Ciências Exatas, Departamento de Ciência da Computação - Belo Horizonte (MG), Brasil.; VIII Universidade Federal de Minas Gerais, Escola de Enfermagem, Departamento de Enfermagem Materno-Infantil e Saúde Pública - Belo Horizonte (MG), Brasil.

**Keywords:** Consumo abusivo de bebidas alcóolicas, Redes generativas adversariais, População sintética, Inquéritos populacionais

## Abstract

**Objetivos:**

Este estudo avaliou o uso de populações sintéticas, geradas por redes generativas adversariais condicionais (cGAN), para estimar o consumo abusivo de bebidas alcoólicas em pequenas áreas de Belo Horizonte (MG).

**Métodos:**

Usou-se dados do Sistema de Vigilância de Fatores de Risco e Proteção para Doenças Crônicas por Inquérito Telefônico (Vigitel) de 2006 a 2018. Foram consideradas variáveis sociodemográficas e de estilo de vida, e os registros sintéticos foram atribuídos a nove *clusters* de vulnerabilidade.

**Resultados:**

A inclusão de dados sintéticos reduziu erros padrão e estreitou intervalos de confiança, sobretudo em áreas mais vulneráveis. Observou-se maior prevalência em áreas menos vulneráveis.

**Conclusões:**

A abordagem mostrou-se promissora para superar limitações de representatividade e ampliar o monitoramento de fatores de risco, mas ainda depende da qualidade dos dados originais, pode introduzir vieses e requer validação externa e uso cauteloso.

## INTRODUÇÃO

A análise de fatores de risco em pequenas áreas é essencial para compreender desigualdades sociais em saúde e para subsidiar o planejamento de políticas públicas locais. O Brasil, apesar de avanços em vigilância, ainda apresenta dificuldades na produção de estimativas válidas em pequenas áreas, em razão dos altos custos e dos desafios na coleta de dados, que inviabilizam a implementação de inquéritos representativos. O Sistema de Vigilância de Fatores de Risco e Proteção para Doenças Crônicas por Inquérito Telefônico (Vigitel), inquérito telefônico anual que abrange as capitais, constitui uma das principais fontes de monitoramento, mas sua dependência de linhas fixas limita a representatividade em grupos mais vulneráveis, como populações de baixa renda e moradores de áreas periféricas^1^.

Essas limitações têm estimulado o desenvolvimento de metodologias alternativas para aumentar a precisão em pequenas áreas. A literatura internacional sugere o uso de abordagens como modelos bayesianos, multinível e técnicas de estimação em pequenas áreas^2^.

Mais recentemente, inovações em inteligência artificial, como as redes generativas adversariais condicionais (cGAN), vêm sendo aplicadas para gerar populações sintéticas realistas, permitindo simulações que ampliam a capacidade de análise em contextos em que os dados reais são escassos, confidenciais ou de difícil obtenção^3^. Este estudo teve como objetivo avaliar o uso de populações sintéticas, geradas por cGAN, para estimar o consumo abusivo de bebidas alcoólicas em pequenas áreas de Belo Horizonte (MG), explorando seu potencial metodológico e discutindo suas implicações.

## MÉTODOS

Trata-se de um estudo ecológico realizado com dados do Vigitel para Belo Horizonte (MG), entre 2006 e 2018. Os endereços dos entrevistados, fornecidos pelas operadoras, foram georreferenciados e vinculados ao Cadastro Nacional de Endereços para Fins Estatísticos, permitindo a associação a setores censitários^2^.

O consumo abusivo de bebidas alcoólicas foi definido pelo consumo de quatro ou mais doses em uma ocasião para mulheres e cinco ou mais para homens^4^.

Consideraram-se como “pequenas áreas” nove *clusters* de vulnerabilidade (BA-0, BA-1, MD-0, MD-1, EL-0, EL-1, EL-2, ME-0, ME-1), derivados das quatro categorias do Índice de Vulnerabilidade à Saúde (IVS) (Baixo, Médio, Elevado e Muito Elevado). Essa estratégia de reagrupamento foi proposta em estudo anterior^5^ para reduzir a variabilidade do IVS. As populações sintéticas foram geradas por redes cGAN implementadas em PyTorch, seguindo a arquitetura clássica de GAN, em que gerador e discriminador são otimizados conjuntamente em cenário adversarial. O treinamento ocorreu por 1.024 épocas, com *batch size* de 500, taxa de aprendizado de 2 × 10⁻^4^ e otimizador Adam (parâmetros padrão). O critério de parada baseou-se apenas no número de épocas, sem *early stopping*, e a função de perda adotada foi a adversarial canônica (*minimax log loss*). As variáveis utilizadas foram sociodemográficas e de estilo de vida (por exemplo, sexo, idade, raça/cor, escolaridade e consumo diário de frutas), selecionadas pela relevância epidemiológica e disponibilidade na base. Foram gerados entre 5 mil e 100 mil registros sintéticos por *cluster*, dos quais subamostras de 700 indivíduos foram selecionadas (definidas com base na estabilização do erro padrão em ~1%) e incorporadas, por modelos supervisionados como o XGBoost, à base do Vigitel nos *clusters* de elevada e muito elevada vulnerabilidade, totalizando 3.500 entrevistas por período (Tabela 1). A similaridade entre distribuições reais e sintéticas foi avaliada pelo índice KSComplement.

A prevalência e os intervalos de confiança de 95% (IC95%) foram estimados por *cluster* e período (2006-2009; 2010-2013; 2014-2018), considerando-se os pesos de pós-estratificação. As diferenças entre *clusters* em um mesmo período foram avaliadas pela não sobreposição dos IC95%.

O estudo foi aprovado pelo Comitê de Ética em Pesquisa da Universidade Federal de Minas Gerais (Parecer n^o^ 6.538.883).

**Tabela 1.** Tamanho da amostra do Sistema de Vigilância de Fatores de Risco e Proteção para Doenças Crônicas por Inquérito Telefônico (Vigitel) e da população sintética por cluster e por período.

Cluster	2006 a 2009			2010 a 2013			2014 a 2018		
	População real	População sintética	Total	População real	População sintética	Total	População real	População sintética	Total
1-BA-0	1.723	-	1.723	1.453	-	1.453	1.570	-	1.570
2-BA-1	1.228	-	1.228	1.112	-	1.112	946	-	946
3-MD-0	1.477	-	1.477	1.297	-	1.297	1.485	-	1.485
4-MD-1	1.547	-	1.547	1.628	-	1.628	1.721	-	1.721
5-EL-0	324	700	1.024	356	700	1.056	376	700	1.076
6-EL-1	130	700	830	158	700	858	197	700	897
7-EL-2	601	700	1.301	683	700	1.383	763	700	1.463
8-ME-0	93	700	793	104	700	804	107	700	807
9-ME-1	199	700	899	242	700	942	244	700	944
Total	7.322	3.500	10.822	7.033	3.500	10.533	7.409	3.500	10.909

## Declaração De Disponibilidade De Dados:

Dados disponíveis mediante solicitação ao pesquisador correspondente.

## RESULTADOS

As prevalências de consumo abusivo de bebidas alcoólicas foram consistentemente mais elevadas nos *clusters* de menor vulnerabilidade, em todos os períodos analisados, tanto nos dados reais (T1 28,2%; IC95% 25,1-31,3; T2 26,2%; IC95% 23,0-29,4; T3 27,0%; IC95% 22,9-31,0) quanto nos sintéticos (T1 27,4%; IC95% 24,7-30,0; T2 27,7%; IC95% 24,7-30,7; T3 26,5%; IC95% 22,6-30,5) (Tabela 2).

**Tabela 2.** Prevalência de consumo abusivo de bebidas alcoólicas nos nove *clusters de* vulnerabilidade para os períodos 2006 a 2009 (T1), 2010 a 2013 (T2) e 2014 a 2018 (T3).

	População real						População real+sintética					
	T1 % (IC95%)	EP	T2 % (IC95%)	EP	T3 % (IC95%)	EP	T1 % (IC95%)	EP	T2 % (IC95%)	EP	T3 % (IC95%)	EP
BA-0	23,5 (21,0-25,9)	1,2	23,9 (21,2-26,7)	1,4	24,1 (21,2-26,9)	1,5	23,3 (21,12-25,4)	1,1	24,1 (21,51-26,6)	1,3	24,0 (21,3-26,7)	1,4
BA-1	28,2 (25,1-31,3)	1,6	26,2 (23,0-29,4)	1,6	27,0 (22,9-31,0)	2,1	27,4 (24,7-30,0)	1,4	27,7 (24,7-30,7)	1,5	26,5 (22,6-30,5)	2,0
MD-0	20,3 (17,7-22,9)	1,3	20,7 (18,0-23,4)	1,4	21,7 (18,9-24,5)	1,4	19,5 (17,3-21,6)	1,1	21,8 (19,3-24,3)	1,3	21,7 (19,1-24,3)	1,3
MD-1	20,5 (18,0-22,9)	1,2	18,9 (16,7-21,2)	1,1	20,9 (18,3-23,4)	1,3	20,1 (17,9-22,3)	1,1	19,1 (17,0-21,1)	1,1	20,6 (18,1-23,0)	1,2
EL-0	11,2 (7,4-14,9)	1,9	18,4 (13,5-23,3)	2,5	18,3 (13,1-23,5)	2,6	12,0 (9,8-14,2)	1,1	17,5 (14,8-20,2)	1,4	18,9 (15,9-21,8)	1,5
EL-1	17,2 (9,3-25,1)	4,0	19,6 (11,8-27,5)	4,0	21,6 (14,4-28,9)	3,7	17,8 (14,9-20,6)	1,5	19,1 (15,9-22,3)	1,6	21,3 (18,0-24,6)	1,7
EL-2	17,0 (13,3-20,7)	1,9	15 (11,6-18,4)	1,7	15,2 (11,8-18,5)	1,7	17,6 (15,3-19,9)	1,2	14,6 (12,4-16,8)	1,1	15,2 (12,9-17,5)	1,2
ME-0	15,9 (7,3-24,4)	4,3	20,3 (10,3-30,3)	5,1	9,5 (3,2-15,7)	3,2	15,3 (12,4-18,2)	1,5	20,6 (17,3-23,9)	1,7	9,2 (6,7-11,7)	1,3
ME-1	14,5 (8,8-20,2)	2,9	14,0 (9,2-18,8)	2,4	15,2 (9,3-21,1)	3,0	14,3 (11,7-16,9)	1,3	14,9 (12,1-17,7)	1,4	15,2 (12,3-18,0)	1,5

EP: erro padrão; BA: baixo; MD: médio; EL: elevado; ME: muito elevado; IC95%: intervalo de confiança de 95%; KSComplement: 0.96.

A inclusão de populações sintéticas na base reduziu os erros padrão e estreitou intervalos de confiança, principalmente nas áreas mais vulneráveis, onde as amostras originais eram menores. Por exemplo, no *cluster* ME-0 e período T1, o IC95% variou de 7,3-24,4% (EP=4,3) com dados reais e de 12,4-18,2% (EP=1,5) com dados sintéticos (Tabela 2). O índice KSComplement de 0,96 confirmou alta similaridade entre as distribuições observadas e sintéticas.

## DISCUSSÃO

Os achados reforçam que o uso de populações sintéticas geradas por cGAN pode mitigar limitações de inquéritos de base populacional ao produzir estimativas mais precisas em pequenas áreas. O maior consumo de bebidas alcóolicas em áreas menos vulneráveis está em consonância com a literatura nacional e internacional^4^ ^,^ ^6^ e pode ser explicado pelo fato de que, em contextos de maior renda, há não apenas maior disponibilidade financeira para a aquisição de bebidas alcoólicas, mas também maior oferta de estabelecimentos comerciais e espaços de socialização que estimulam o consumo. Além disso, aspectos culturais e comportamentais, como a valorização de práticas sociais vinculadas ao uso de bebidas alcoólicas, contribuem para reforçar esse padrão. Por outro lado, áreas mais vulneráveis podem apresentar menor prevalência relatada, mas estão mais expostas a agravos decorrentes do consumo, pela menor disponibilidade de serviços de saúde e suporte social.

A utilização de dados sintéticos contribuiu para maior estabilidade das estimativas, reduzindo a variabilidade entre períodos e mostrando-se uma abordagem promissora para mitigar a escassez de dados coletados e reduzir vieses de distribuição e representação em pequenas áreas geográficas.

Apesar dos avanços metodológicos, a técnica deve ser considerada exploratória. Sua aplicação depende da qualidade dos dados reais, pode introduzir vieses relacionados à modelagem e exige validação externa em diferentes contextos^7^. Questões éticas emergem do uso de dados sintéticos, incluindo a necessidade de transparência e de salvaguardas contra usos inadequados. Quando comparadas a abordagens tradicionais de pequenas áreas, como modelos bayesianos e multinível, as cGAN apresentam desempenho competitivo e complementar^2^ ^,^ ^7^. No entanto, sua adoção requer estudos adicionais que avaliem sensibilidade, generalização e replicação em outras populações.

Assim, conclui-se que populações sintéticas representam um recurso inovador para vigilância em saúde, mas seu uso deve ser acompanhado de avaliações críticas, validação contínua e reflexão ética. Essa inovação pode apoiar a formulação de políticas públicas mais precisas e equitativas, desde que utilizada de forma responsável.
